# Physiological and Molecular Mechanism Involved in Cold Stress Tolerance in Plants

**DOI:** 10.3390/plants9050560

**Published:** 2020-04-28

**Authors:** Faujiah Nurhasanah Ritonga, Su Chen

**Affiliations:** State Key Laboratory of Tree Genetics and Breeding, Northeast Forestry University, Harbin 150040, China; ritongafaujiah@ymail.com

**Keywords:** chilling, cold acclimation, freezing, low temperature, ICE-CBF-COR, tolerance

## Abstract

Previous studies have reported that low temperature (LT) constrains plant growth and restricts productivity in temperate regions. However, the underlying mechanisms are complex and not well understood. Over the past ten years, research on the process of adaptation and tolerance of plants during cold stress has been carried out. In molecular terms, researchers prioritize research into the field of the ICE-CBF-COR signaling pathway which is believed to be the important key to the cold acclimation process. Inducer of CBF Expression (*ICE*) is a pioneer of cold acclimation and plays a central role in C-repeat binding (CBF) cold induction. *CBFs* activate the expression of *COR* genes via binding to cis-elements in the promoter of *COR* genes. An ICE-CBF-COR signaling pathway activates the appropriate expression of downstream genes, which encodes osmoregulation substances. In this review, we summarize the recent progress of cold stress tolerance in plants from molecular and physiological perspectives and other factors, such as hormones, light, and circadian clock. Understanding the process of cold stress tolerance and the genes involved in the signaling network for cold stress is essential for improving plants, especially crops.

## 1. Introduction

Drought, salinity, and low temperature (LT) are the main abiotic stress factors that have strong impacts on plant growth and development [1]. In addition to drought stress, LT is one of the most harmful environmental stresses encountered by higher plants [2,3]. LT stress is divided into chilling stress (<20 °C) and freezing stress (<0 °C) according to the environmental temperature. In addition to affecting the growth and development of the plant, LT stress significantly restrains the geographical distribution of plants [4,5,6,7]. 

Tropical and subtropical plants are sensitive to chilling stress and lack the capacity of cold acclimation. However, temperate plants have the ability to tolerate freezing temperatures following a period of exposure to non-freezing temperatures, which is termed as cold acclimation [8]. Temperate plants are tolerant to seasonal changes in temperature and can tolerate cold stress during early spring and winter. Many important crops, such as rice, corn, soybean, potato, cotton, and tomato, are chilling sensitive and incapable of cold acclimation. In contrast, some crops, such as oats, are chilling tolerant but freezing sensitive. On the other hand, barley, wheat, and rye are well adapted in freezing temperatures [9]. However, some plants such as *Arabidopsis*, winter wheat (*Triticum aestivum* L.) and barley (*Hordeum vulgare* L.) are not able to tolerate nonfreezing temperatures without cold acclimation [10]. Similarly, *Santalum album* and *Betula utilis* (Himalayan Birch), as dominant vegetations in cold environments (Eastern Nepal), are also sensitive to cold during the growing season [11,12]. 

Each plant has different enrichment pathways in different periods of cold stress, such as the amino sugar and nucleotide sugar metabolism pathway, alanine and protein export, and the aspartate and glutamate metabolism are highly enriched during the latter stages of cold stress periods in tea plants. In contrast, cellular components, biological processes, and the molecular function category are highly enriched in the early periods of cold stress in tea plants. The same result was found using transcriptome analysis in which flavonoid biosynthesis, phagosome, plant hormone signal transduction, and fructose were highly enriched at the beginning of cold stress [13]. In tomato, even though the expression of ethylene signaling genes decreased after cold stress, none of the protein genes decreased. This shows the different regulation of gene and protein levels [14]. In addition, abscisic acid (ABA) signaling pathway gene expression improved the cold hardiness in grapevine buds only during the cold acclimation period [15]. Furthermore, the overexpression of *GLR1.2* or *GLR1.3* enhanced cold tolerance by increasing endogenous jasmonate levels under cold stress. However, they could not interact with each other directly, indicating they interact indirectly to achieve cold tolerance [16]. In banana and *Zoysia japonica*, *MaPIPI2.7* and *ZjICE1* improved multilevel stresses, such as cold, salt, and drought stress [17]. These results show that every gene has a different mechanism in different plants to achieve abiotic or biotic stress tolerance. Due to these differences of signal transduction pathways and metabolisms, the mechanisms related to cold stress are complex [18].

The methods plants use to deal with adverse environmental stress, including stress avoidance and stress tolerance, have been examined in the literature [19,20]. Recent studies of LT stress and cold acclimation based on the model plant *Arabidopsis* contribute substantially to understanding the molecular mechanisms of cold acclimation [6]. In addition, numerous studies have been carried out in other plant species to reveal the molecular mechanism and gene regulatory networks. In this review, we aim to give a comprehensive overview of the current knowledge about plant under LT stress.

## 2. Genetical Changes during Cold Stress

Investigation of transcriptional alterations of plants during cold acclimation is crucial for understanding the underlying molecular mechanism under LT stress. Until now, a significant number of cold responsive genes have been identified and several gene regulated networks have been reported. Among these, ICE-CBF-COR is one of the most wildly reported pathways. In most plant species, the ICE-CBF-COR pathway is induced by LT stress and then activates the appropriate expression of downstream genes, which encode osmoregulation substances [9]. Inducer of CBF Expression (*ICE*) is a pioneer of cold acclimation, an MYC-type basic helix-loop-helix family transcription factor (TF) [8,21,22]. It is reported that *ICE1* plays a central role in C-repeat binding factor 3 (*CBF3*) cold induction. When plants encounter LT stress, *ICE1* could be released from JAZs bound by DELLAs and induce the expression of *CBF3*. *CBF3* activates the expression of *GA2ox7* to reduce the bioactive gibberalic acid (GA) level, which promotes the accumulation of DELLAs. Therefore, DELLAs can regulate the cold induction of *CBF3* through *ICE1* via JAZs. 

**Figure 1:**
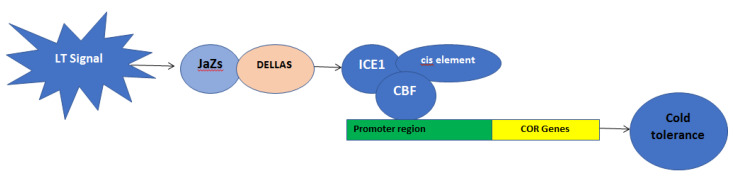
ICE-CBF-COR pathways in plants tolerance to cold stress. The expression of *CBFs* is mainly mediated by DELLA signaling and induced by *ICE1*. DELLAs contribute to the cold induction of *CBF* genes through interaction with JaZs signaling. *CBFs* activate the expression of *COR* genes via binding to cis-elements in the promoter of *COR* genes and result in the enhancement of cold tolerance in plants.

C-repeat binding factors (*CBFs*), also known as dehydration-responsive element-binding proteins (DREBs), act as a regulating gene that has an important role in cold acclimation [23]. CBF is a member of the APETALA2/ETHYLENE RESPONSE FACTOR (AP2/ERF) family and regulates the expression of the cold-responsive (*COR*) gene [24]. The AP2/ERF family is one of the largest TF families in plants and is characterized by having a minimum of one AP2 DNA-binding domain [25]. *CBFs* activate the expression of *COR* genes via binding to cis-element in the promoter of *COR* genes (CRT: TGGCCCGAC) (Figure 1) [23,26]. In addition to LT stress, CBFs also play important roles in other abiotic stresses [7,26,27]. Some of the cold-responsive genes have an ABA responsive element and dehydration responsive element in their promoter regions [28]. *EgDREB1* from oil palm might also have a similar regulatory element located in a sequence promoter and are responsive to cold signaling [29]. It was also found in *DREB* [30] and *FtbHLH2* that the transgenic plant promoter was induced by cold and that many cis-elements of the *FtbHLH2* promoter collaborate in cold stress conditions [31].

In temperate plants of wheat and barley, *CBFs* are found in the Frost Resistance 2 (FR2) locus [4]. Winter barleys planted in early autumn need collaboration between Vernalization 1(*VRN1*) and *CBF* genes to enhance cold acclimation. However, for spring barley, vernalization is not required. As a result, spring barley is not freezing tolerant. Similar to spring barley, 214 upregulated genes and 884 downregulated genes were found in grapevine cultivars after LT treatment [25], suggesting that cold stress induced the downregulated genes. This study found that *CBF1*, *CBF2*, and *CBF3* had no role in cold acclimation in grapevine leaves. This is due to the absence of *CBF1* and low levels of *CBF2* and *CBF3*. However, there were some exceptional cases in the mechanisms of the *CBF* gene family expression. A study found that *ICE1* mutation only had a small effect on the *CBF* transcript [21]. The expression of *CBF1* is also regulated by the SVALKA-CBF1 cascade. *SVALKA* is a long non-coding RNA (lncRNA) that is transcribed by RNA polymerase II (RNAPII). Mutation in *SVALKA* affects expression of *CBF1* and plant freezing tolerance. The SVALKA-CBF1 regulatory network has been found in species other than *Arabidopsis* [32]. 

The implementation of CBF-regulated genes is not only for cold stress but also for salt stress, hormone response, and even carbohydrate metabolism [33]. Previous research found that at 3 h or 24 h of cold treatment in *Arabidopsis cbfs* triple mutant, the number of up-regulated genes was 609 and 1375, respectively, and the number of down-regulated genes was 163 and 1349, respectively [33]. In addition, a total of 1394 cold-induced genes and 1113 cold-repressed genes were recognized in both the wild-type and *cbfs* triple mutant. This indicates that a large number of *COR* genes are not influenced in the *cbfs* triple mutant within cold stress. 

In addition, numerous TFs that can regulate cold signaling and cold stress have been identified, such as *CBF1, CBF2, CBF3, ICE1, ICE2, CAMTA3, MYB15, ZAT12, COR15a*, and *COR15b* [34]. Some TF families found to be related to LT stress in *Pyrus ussuriensis* are DREB, WRKY, NAC, MYB, AP2/ERF, and bHLH [35]. Brassinosteroids are plant hormones that have an important role in plant growth and can also protect plants against abiotic stress such as cold stress. Two Brassinosteroids (BRs) TFs, namely, Brassinazole-resistance 1 (*BZR1*) and CESTA (*CES*), are direct regulators of *CBF* [36,37].

Several factors influence the binding affinities of TFs to specific sites, such as chromatin accessibility, DNA methylation, TF cooperativity, and TF interactions with non-binder cofactors and the transcription machinery [38]. A single TF regulates the expression of many downstream genes, so the utilization of TFs proffers many advantages in genetic engineering [39]. Some protein kinases (MEKK1-MKK1/2-MPK4) induce the expression of *CBFs*, especially *CBF2* [6,40]. Another gene that also plays a role in the cold acclimation process is Cold Induced Small Protein 1 (*CISP1*), which was found in the roots of the *Poaceae* plant (a case study on barley). Normally, *CISP1* is increased after 27 days of low-temperature treatment. Homologous adherents of *CISP1* (i.e., *CISP2* and *CISP3*) are also found in several *Poaceae*, and it also plays a role in the cold acclimation process [41].

Overexpression of *FtbHLH2* in the transgenic plant increases the expressional level of *CBF1*-*3* and enhances low temperature tolerance in plants [31]. Other studies suggested that ethylene response factors from *Vitis amurensis*, *VaERF080,* and *VaERF087* (AP2/ERF Family) regulate the expressional levels of cold-related genes including *CBF1*, *CBF2*, *ICE1*, *ZAT12*, *KIN1*, *SIZ1*, *RD29A*, *COR15A* and *COR47* [42]. When treated by cold stress, *CBF1-8* of *Brachypodium distachyon*, a herbaceous grass species that can tolerate cold stress, was upregulated at a different time [43]. 

NADP-dependent D-sorbitol-6-phosphate dehydrogenase (*S6PDH*), anthocyanidin synthase (*ANS*), and phenylalanine ammonia-lyase (*PAL*) genes might play a vital role in the cold response of the loquat [44]. Studies reveals that the expression levels of *S6PDH*, *ANS*, and *PAL* are upregulated by cold treatment during the first 4 h but suppressed as the stress continues. A previous study shows that *SiDHN* found in *Saussurea involucrata* plays a pivotal role in low temperature and drought stress. After being grown under a low-temperature treatment for 24 h, the expression level of *SiDHN* is increased three-fold. This proved that *SiDHN* is responsive to cold stress [5]. Recently, *Arabidopsis* overexpressing RNA-DIRECTED DNA METHYLATION 4 (*RDM4*) showed the antagonist result with the *rdm4* mutant in facing cold stress. Overexpression of *RDM4* in plants increases the expression of *CBFs* and the downstream genes after chilling stress, subsequently improving the survival rate. However, while the *rdm4* mutant shows an increase of electrolyte leakage and H_2_O_2_ content, the survival rate decreased after chilling treatment [45]. In *Arabidopsis*, *RDM4* promotes the affinity of polymerase II (Pol II) to the promoter of *CBF* genes and, as a result, increases the cold tolerance of *Arabidopsis* [45].

In rice (*Oryza sativa*), during the recovery period from cold stress, transgenic lines overexpressing *OsGH3-2* exhibit enhanced cold tolerance compared to wild type plants. Even after 7 days of recovery, more than 80% of the transgenic plants remain vigorous, whereas almost all wild type plants died [46]. Another study shows that *OsMADS57* and *OsTB1* are directly targets of *OsWRKY94* and axillary bud regulated gene *D14* during cold adaptation in rice. This provides evidence that *OsMADS57* acts as a molecular linker between the developmental response and the tolerance to chilling stress in rice [47]. After the recovery process, *OsMADS57* could still preserve cell division during low temperatures [47]. Transgenic tobacco overexpressing *GhDREB1* showed improved tolerance to chilling stress compared to wild plants in early seedling and later seedling stages. *GhDREB1* was also detected as a transcriptional activator of *NtERD10B* and *NtERD10C* after cold stress treatment in transgenic tobacco [48]. A gene that is likely responsible for cold stress was also found in *Sorghum bicolor* [49]. The results were obtained through significant regions, proxies, or co-localization with single nucleotide polymorphisms (SNPs) and also on homology with photosynthesis and stress-responsive genes. The gene is a *GST* gene family, namely *SB08g007310* (Table 1). The gene function is also related to photosynthesis, and carbon and nitrogen metabolism [49]. Ectopic expression of a CBF pathway independent chilling tolerance gene (*AtGRXS17*) in tomato enhances chilling tolerance of tomato via collaboration with *CBFs* [50]. In recent years, transcriptome and bioinformatics has been increasingly used to address complex biological questions [51,52,53,54,55] and more cold stress related genes will be investigated. Moreover, a large number of mutant lines have been developed for the functional study of the genes in the plant genome, including those inserted by Transfer DNA (T-DNA) and RNA Interference (RNAi) [51,52]. In addition, Clustered Regularly Interspaced Short Palindromic Repeats (CRISPR)/CRISPR-associated protein 9 (Cas9), also known as a genome editing tool, allow scientists to change the DNA of an organism. CRISPR/Cas9 has been widely used, since it can be successfully used to edit multiple genes in a plant [56,57,58,59]. However, the lack of sufficient genomic sequence information in many plants is a limitation of CRISPR/Cas9. In the future, it would be prudent to develop this technology’s ability to elucidate multiple genes, TFs, and protein functions in plants that are yet to be identified and may have a role in cold stress. 

## 3. Physiological Changes during Cold Stress

A large number of plant species display physiological or cellular perturbations when encountering LT stress. Under LT stress, plants need to maintain cell behavior and activity, and, in particular, the stability of the cell membrane and structure of the protein with biological activity, for survival in adverse environments [47].The exposure of plants to subzero temperature leads to ice formation in plant tissues [74]. Higher concentrations of active ice nucleators in the apoplastic solution of plants leads to a higher freezing point. As a result, ice crystals first form in the extracellular space of plant cells. Ice formation outside cells reduces the water potential of the apoplastic solution, which leads to water flowing from the cells. Therefore, freezing stress at the cellular level is often followed by dehydration stress. Ice crystals will lead to an increase in electrolyte leakage and membrane lipid phase changes. As the freezing continues, osmotic forces produce cellular dehydration, which facilitates the formation of intracellular ice crystals. At the extreme, ice crystals can puncture plant cells and lead to cytosol outflow, and ultimately cause the plants to die [9,42,75]. Therefore, preventing formation of intracellular ice crystals and avoiding growth of ice crystals are important for plants to tolerate cold stress. The most popular approach used by plants to deal with LT stress is cold acclimation, which allows plants to survive freezing via accumulation of cryoprotective polypeptides (e.g., *COR15a*) and osmolytes (e.g., soluble sugars and proline). It has been reported that sugar content in *Euphorbia resinifera, Echinocactus grusonii, Aloe vera*, *Crassula lacteal, Bryophyllum pinnatum, Yucca aloifolia,* and *Sansevieria trifasciata* is increased after cold stress [76]. Cold adaptive plants always store more sugar (D-Glucose, D-Glucose 6 Phosphate, amylose, starch, and maltose) in their underground tissues [25,77].

A recent study of grapevine reveals that grapevine leaves have a watery appearance, indicating tissue damage and cell leakage after freezing treatment. After 4 days of recovery, damage was found in the leaves [25]. Necrosis of plants is normally caused by overproduction of Reactive Oxygen Species (ROS). The elevated H_2_O_2_ level in plants under LT stress is the result of increased oxygenation reaction in the chloroplasts, which leads to an increased glycolate content. The glycolate is converted to glyoxylate in peroxisomes by glycolate oxidase, which is accompanied by accumulation of H_2_O_2_. The physiological process of ROS toxic concentration in plants could be relieved by developing a complicated and efficient ROS scavenging and antioxidant defense system. Plants require the use of low ROS concentrations as mediators for signal transduction. Nitric oxide (NO) is implicated in the response of plants to LT stress. ABA, Ca^2+^, and H_2_O_2_-associated NO are shared by signaling cold stress events [78]. Hemoglobin (Hb) is believed to be a modifier of low-temperature plant response through the transition of NO [79]. Hb over-expressing lines have demonstrated reduced cold-induced gene expression. However, a decrease was only seen in *CBF1* and *CBF3*, not *CBF2* [80]. Moreover, an increased malondialdehyde (MDA) content and Ca^2+^ in the cytosol characterized lipid degradation, while the activity of antioxidant enzymes, such as Catalase (CAT), superoxide dismutase (SOD), ascorbate peroxidase (APX), and peroxidase (POD), was minimized [22,42,81,82]. In addition, ABA and ROS can induce Ca^2+^ and will affect cold signaling [8].

After methyl jasmonate (MeJa) treatment in bell pepper [34] and ethylene treatment in *Arabidopsis* [42], bell pepper and *Arabidopsis* are more tolerant to cold stress and chilling stress. Their physiological function is changed, namely, via decrease of MDA content and increase of antioxidant enzyme activities, such as CAT, SOD, and POD. This proves that these plants have a high tolerance to cold stress [42]. Other research about cold stress revealed that cold could induce the inhibition of CO_2_ assimilation in *Zea mays* and was also related to a persistent depression of the photochemical efficiency of PSII [83]. Moreover, NO is known as a signal in an early transduction network. Cold triggers NO a few hours after exposure to low temperature in *Arabidopsis* [84]. Arabidopsis hemoglobin 1 (*AHb1*) interferes with NO production before and after exposure to low temperature. However, *A. thaliana* over-expressing *AHb1* resists its capacity to produce NO during cold stress [84].

Nevertheless, lowering the temperature decreased the translocation of xylem N in *S. cereale* and *B. napus* by about 60% and 30%, respectively. It is suggested that low temperatures will directly affect the absorption mechanism of nitrates and N accumulation in the roots. This is caused by an obstruction of the N xylem flow greater than the NO absorption [85]. 

LT also contributed to decreasing chlorophyll content of rice. Putting rice at low temperatures modified the number of chloroplasts, the arrangement of grana (no normal stacked membranous structures), and the lamellar structures in chloroplasts. In addition, temperature-sensitive virescent (TSV) could improve the stability of *OsTrxZ* at low temperatures, which is critical to the production of chloroplasts during or after cold stress in *Oryza sativa* [86]. This is due to the abnormal development of chloroplasts during and after low-temperature stress [87]. 

## 4. Influence Factors 

Plants can sense several parameters of light signals, such as light quality (wavelength), quantity, and duration (daylight), and even direction. The length of the daily photoperiod and moderate subfreezing temperature greatly affects the hardening and dehardening processes in Scots pine [81]. Short day (9 h) and long day (16 h) first frost temperatures are different with 16 h and 9 h daily photoperiod: the first frost is at about −10 °C and frozen at −22 °C. This means that light has a pivotal role in the hardening process. A previous study suggested that light is one of the regulators of *FDA2-3* and *FDA2-4* gene expression in cotton (*Gossypium hirsutum*). Light and low temperature induces the expression of these two genes and allows the plant to tolerate cold stress [30]. Meanwhile, LT reduces the utilization of light [82]. Light has a pivotal effect on the relationship between insects and plants, while temperature affects light performance [88]. However, the light also acts as an external signal that influences the growth of the plant [89].

**Figure 2:**
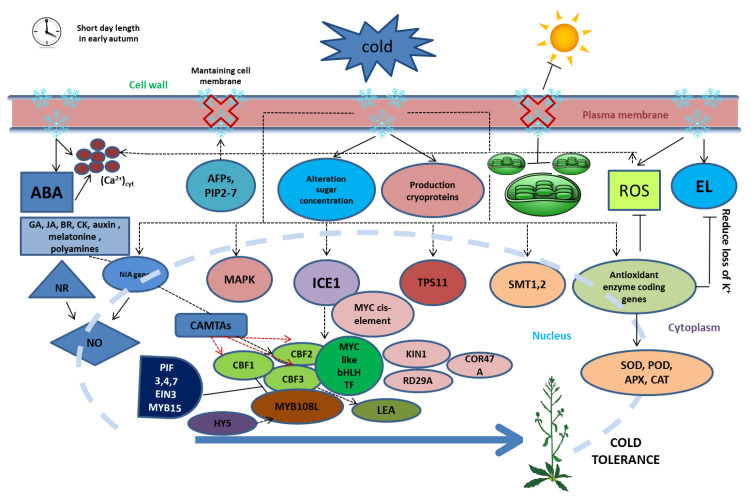
The mechanism of cold tolerance in plants. A short day in early autumn represents the first initiation of cold stress. AFPs and PIP2-7 slow ice crystal formation to maintain cell membranes and reduce membrane injury. However, low temperature (LT) still causes some changes in membrane structure, sugar concentration, and production in cry proteins. LT initiates the increase of ABA, EL, (Ca^2+^)_cyt_, and ROS accumulation but decreases chloroplast number. LT induces the expression of some genes, such as *NIA*, *MAPK*, *TPS11*, *SMT1*,2, *ICE1* and antioxidant enzyme coding genes. Antioxidant enzyme coding genes reduce the EL and increase the activity of the antioxidant enzymes in cold stress plants. Meanwhile, *NIA* genes and NR initiate NO as a result of LT. *ICE1*, CAMTAs, *NIA*, and hormones induce the expression of *CBFs*, which bind to CRT/DRE cis-elements to enhance cold tolerance. ICE1-CBFs induce expression of cold-responsive genes, such as *KIN1*, *RD29A*, *COR47A*, and *LEA*, during cold stress.

Similar to light, hormones also have a role in the activation process of TFs [35,37]. Although the ICE-CBF-COR pathway plays a key role in the cold tolerance in plants, researchers are currently focusing on the contribution of hormones to cold stress. Major hormones, such as ABA, GA, brassinosteroids (BR), jasmonates (JA), auxin, cytokinin (CK), melatonin, and polyamines, affect *CBFs* (Figure 2) [37]. One of the most important hormones in cold stress is ABA [90]. ABA increases when stress surge affects plants and, contrary to ABA, the TF is not affected. Furthermore, an overlapping linkage between the ABA-dependent pathway and the ICE-CBF-COR pathway was found in cold tolerance [37]. A number of studies suggest that ABA can induce increases in the transcript levels of *CBF* genes, perhaps via binding to the CRT/DRE element. It has the potential to encourage *CBF* activation. In addition, *AtHAP5A* regulates freezing stress tolerance through binding to the CCAAT motif of *AtXTH21* in *Arabidopsis*. *AtHAP5A* and *AtXTH21* overexpressing plants were more tolerant of freezing stress but less susceptible to ABA than WT plants [61]. Moreover, ABA also plays an important role in the acclimation process and can induce *COR* gene expression [26]. Some genes in *Pyrus ussuriensis* also collaborate with ABA, Gibberellin, and Ethylene to up-regulate genes [35]. The ABA signal transduction pathway also has a positive regulator, namely, the *BpUVR8* gene, which regulates the expression of a subset of ABA-responsive genes, both in *Arabidopsis* and *Betula platyphylla* under ABA treatment [60]. ABA pretreatment was also successful in increasing the mechanism of cold stress tolerance in the root level of *Brassica rapa* with phase <3 h and decreased the deleterious effect of Paclobutrazol (PBZ), which reduced the root hydraulic conductance of *B. rapa* [91].

Other studies have found plant mutants with altered levels of GA. GA will increase rice tolerance in stress. In research of *B. napus*, the gibberellin 2 oxidase gene was added into a transgenic plant and caused dwarfism. In comparison to the WT plants, the dwarf transgenic plants were 18.3–26.1% shorter in height and had 17.8–33.6% shorter internodes, and smaller and dark green leaves. GA and JA play an efficient role in the ICE-CBF-COR pathway [37]. Jasmonates activate TFs and then TFs will bind with the cis-acting element in the promoter of target genes [92].

Ethylene, ABA, and Jasmonates hormones can induce the expression of ethylene-responsive (*ERF*) genes [93]. These genes can be found in species such as *Arabidopsis* (*AtERF6*), *Citrus sinensis* (CsERF), *Lycopersicon esculentum* (*LeERF3b; SIERF5*), *Nicotiana tabacum* (*JERF1; JERF3*), *Triticum aestivum* (*TaERF1*), and *Triticum turgidum* (*TdSHN1*). At low temperature, *ERFs* will bind with the GCC box and DRE elements and provide some plants with tolerance to cold stress. 

In addition, the *BpGH3.5* gene also affects Indole-3-acetic acid (IAA) in birch. This is shown by the decrease of auxin, which is indicated by a decrease in IAA. Some research results show that GH3 gene families play an important role in plant growth and development, and the short root phenotype in transgenic birch is caused by changes in IAA levels [94]. In another study, it was also mentioned that *BpGH3.5* causes primary short and lateral roots and more root hairs. More root hairs are caused by increased surface area for nutrient and water uptake, and finally lead to short root length (root dwarfism) [95].

Transgenic plants produce more anthocyanins in winter. It is well known that the anthocyanin content in plants will be higher in cold stress conditions. This means that the Gibberellin 2-oxidase gene perhaps has an important role in cold stress [96]. Carbon ion beam irradiation is one of the tools that can upregulate the expression levels of *CBFs*, *ICE1, ICE2, CAMTA3*, and *COR* genes in cold stress [22]. In addition, melatonin is also known as an influencer of the increase of the levels of mRNA, such as *COR15a* [37]. Expression of those genes is increased by 50 Gy of carbon ion beam irradiation for 6 h and 12 h at 4 °C. Nonetheless, in *Arabidopsis*, carbon ion beam irradiation of 50 Gy increases the content of AsA and GSH, which play important roles in alleviating ROS generation and oxidative stress under cold stress [22]. The level of soil moisture and stomatal conductivity has been determined by Rihan et al. [26]; these two factors can up-regulate the *CBF/DREB1* gene in cauliflower (*Brassica oleracea* var. botrytis). In fact, the addition of methyl jasmonate to bell pepper can induce POD, CAT, and APX relative gene expression. These genes will reduce the ROS content, so the plant will tolerate chilling stress [34].

In addition to the factors noted above, the circadian clock also plays an important role in cold stress [97]. Circadian clocks are original timekeeping networks that authorize organisms to align their physiology processes with environmental changes at relevant times of the day and year. Recently, researchers have found that there are tissue-specific clocks in the plant’s body. However, further research is still needed to discover how the plant tissues are organized and communicate with each other [97]. Geographical location (latitude) and mutation affect the expression of *CBF1* and *CBF2* in *A. thaliana*. When tested under cold treatment, northern areas tend to have higher rates of survival than southern areas [23]. 

## 5. Conclusions

Cold tolerance in plants is a complex process. Chilling or freezing temperatures can trigger the formation of ice in plant tissues, which causes cellular dehydration. On the other hand, plants can protect their body by preventing ice formation. As a complex process, the mechanism of cold stress in plants is not only controlled by ICE-CBF-COR genes but also other factors, such as hormones, circadian clocks, and light. *ICE* is a CBF regulator, and *CBFs* control the expression of the *COR* gene when cold stress takes place. The *COR* gene is a critical gene that is responsible for chilling tolerance and cold acclimation processes in plants. Under normal conditions, *CBFs* are regulated by the circadian clock and the photoperiod. Under cold stress conditions, however, *CBFs* induce several cold stress-related genes to regulate the cold tolerance of plants.

## Figures and Tables

**Table 1:** Cold stress-related genes in plants and their expression under different type of stress conditions.

Gene Name	Family	Species	Type of Stress Condition	References
*FtbHLH2*	bHLH	*Fagopyrum tataricum*	Cold stress	[31]
*BpUVR8*	UVR	*Betula platyphylla*	ABA response and cold stress	[60]
*FDA2-3*	FDA	*Gossypium hirsutum*	Cold stress	[30]
*FDA2-4*				
*FDA8*		*Arabidopsis thaliana*	Cold stress	[30]
*Sb08g007310*	GST	*Sorghum bicolor*	Cold stress	[49]
*Sb06g018220*	ZEP	*Sorghum bicolor*	Epoxidation of zeaxanthin in the xanthophyll cycle	[49]
*AtGRXS17*	Trx	*Solanum lycopersicum*	Chilling stress	[50]
*AtCBF3*	AP2/ERF	*Arabidopsis*	Cold Stress	[50]
*VaERF080*	AP2/ERF	*Vitis amurensis*	Cold stress	[31]
*VaERF087*				
*SiDHN*	DHN	*Saussurea involucrata*	Freezing stress and drought stress	[5]
*OsGH3-2*	GH3	*Oryza sativa*	Drought and cold stress	[46]
*MYBS3*	MYB	*Oryza sativa*	Cold stress	[4]
*RDM4*		*Arabidopsis*	Cold stress and freezing stress	[45]
*OsMADS57*		*Oryza sativa*	Chilling stress	[47]
*GHDREB1*	DREB	*Gossypium* *hirsutum*	Chilling stress	[48]
*AtHAP5A, AtXTH21*		*Arabidopsis thaliana*	Freezing stress	[61]
*PUB25/26*		*Arabidpsis thaliana*	Freezing stress	[62]
*MaPIP2-7*	AQP	*Musa acuminata*	Drought, cold and salt stress	
*MaPIP2-7*	AQP	*Musa acuminata*	Drought, cold and salt stress	[17]
*CsCPKs*	CPK	*Camellia sinensis*	Cold tolerance	[63]
*COR413*	COR	*Saussurea involucrata*	Cold and drought tolerance	[64]
*SET, JmJC*		*Brassica rapa*	Heat and cold stress	[65]
*TaTPS11*		*Triticum aestivum*	Cold stress	[66]
*TaSMT1, TaSMT2*		*Triticum aestivum*	Cold stress	[67]
*14-3-3ε, 14-3-3ω*		*Arabidopsis thaliana*	Cold and oxidative stress	[68]
*CsLEA*	LEA	*Camellia sinensis*	Cold and dehydration stress	[69]
*MdMYB108L*	MYB	*Malus domestica*	Cold stress	[70]
*MdHY5*	bZIP	*Malus domestica*	Cold stress	[70]
*DlICE1*	bHLH	*Dimocarpus longan*	Cold stress	[71]
*ZjICE1*	bHLH	*Zoysia japonica*	Cold, dehydration and salt stress	[72]
*VvCBF*	DREB	*Vitis vinifera*	Cold stress	[15]
*AtGLR1.2* *AtGLR1.3*		*Arabidopsis thaliana*	Cold stress	[16]
*STCH4*		*Arabidopsis thaliana*	Cold stress	[73]
